# Validation of a Standard Protocol to Assess the Fermentative and Chemical Properties of *Saccharomyces cerevisiae* Wine Strains

**DOI:** 10.3389/fmicb.2022.830277

**Published:** 2022-02-23

**Authors:** Patrizia Romano, Gabriella Siesto, Angela Capece, Rocchina Pietrafesa, Rosalba Lanciotti, Francesca Patrignani, Lisa Granchi, Viola Galli, Antonio Bevilacqua, Daniela Campaniello, Giuseppe Spano, Andrea Caridi, Marco Poiana, Roberto Foschino, Ileana Vigentini, Giuseppe Blaiotta, Viviana Corich, Alessio Giacomini, Gianluigi Cardinali, Laura Corte, Annita Toffanin, Monica Agnolucci, Francesca Comitini, Maurizio Ciani, Ilaria Mannazzu, Marilena Budroni, Vasileios Englezos, Kalliopi Rantsiou, Lucilla Iacumin, Giuseppe Comi, Vittorio Capozzi, Francesco Grieco, Maria Tufariello

**Affiliations:** ^1^Faculty of Economy, Universitas Mercatorum, Rome, Italy; ^2^School of Agricultural, Forestry, Food and Environmental Sciences, University of Basilicata, Potenza, Italy; ^3^Department of Agricultural and Food Sciences, Alma Mater Studiorum - University of Bologna, Cesena, Italy; ^4^Department of Agriculture, Food, Environment and Forestry, University of Florence, Florence, Italy; ^5^Department of Agriculture, Food, Natural Resources and Engineering, University of Foggia, Foggia, Italy; ^6^Department of Agriculture, Mediterranea University of Reggio Calabria, Reggio Calabria, Italy; ^7^Department of Food, Environmental and Nutritional Sciences, University of Milan, Milan, Italy; ^8^Department of Agricultural Sciences, Division of Grape and Wine Sciences, University of Naples Federico II, Naples, Italy; ^9^Department of Agronomy, Food, Natural Resources, Animals and Environment, University of Padua, Padua, Italy; ^10^Department of Pharmaceutical Sciences, University of Perugia, Perugia, Italy; ^11^Department of Agriculture, Food and Environment, University of Pisa, Pisa, Italy; ^12^Department of Life and Environmental Sciences, Polytechnic University of Marche, Ancona, Italy; ^13^Department of Agricultural Sciences, University of Sassari, Sassari, Italy; ^14^Department of Agricultural, Forest, and Food Science, University of Turin, Turin, Italy; ^15^Department of Agricultural, Food, Environmental and Animal Science, University of Udine, Udine, Italy; ^16^Institute of Sciences of Food Production, National Research Council of Italy, c/o CS-DAT, Foggia, Italy; ^17^Institute of Sciences of Food Production, National Research Council of Italy, Lecce, Italy

**Keywords:** protocol, validation, *Saccharomyces cerevisiae*, inter-laboratory, intra-laboratory, wine

## Abstract

This paper reports on a common experiment performed by 17 Research Units of the Italian Group of Microbiology of Vine and Wine (GMVV), which belongs to the Scientific Society SIMTREA, with the aim to validate a protocol for the characterization of wine strains of *Saccharomyces cerevisiae*. For this purpose, two commercial *S. cerevisiae* strains (EC 1118 and AWRI796) were used to carry out inter-laboratory-scale comparative fermentations using both synthetic medium and grape musts and applying the same protocol to obtain reproducible, replicable, and statistically valid results. Ethanol yield, production of acetic acid, glycerol, higher alcohols, and other volatile compounds were assessed. Moreover, the Fourier transform infrared spectroscopy was also applied to define the metabolomic fingerprint of yeast cells from each experimental trial. Data were standardized as unit of compounds or yield per gram of sugar (glucose and fructose) consumed throughout fermentation, and analyzed through parametric and non-parametric tests, and multivariate approaches (cluster analysis, two-way joining, and principal component analysis). The results of experiments carried out by using synthetic must showed that it was possible to gain comparable results from three different laboratories by using the same strains. Then, the use of the standardized protocol on different grape musts allowed pointing out the goodness and the reproducibility of the method; it showed the main traits of the two yeast strains and allowed reducing variability amongst independent batches (biological replicates) to acceptable levels. In conclusion, the findings of this collaborative study contributed to the validation of a protocol in a specific synthetic medium and in grape must and showed how data should be treated to gain reproducible and robust results, which could allow direct comparison of the experimental data obtained during the characterization of wine yeasts carried out by different research laboratories.

## Introduction

*Saccharomyces cerevisiae* is the predominant yeast species in winemaking. Due to its adaptability to the stressful conditions imposed by grape must fermentation, it easily competes with other yeasts and bacteria, and being the main actor of the transformation of grape must into wine, it is universally known as the “wine yeast.”

In the last decades, a wide number of molecular and physiological studies demonstrated the high genotypic and phenotypic diversity of *S. cerevisiae* wine strains (Romano et al., 2008; Csoma et al., 2010; Mercado et al., 2011; Capece et al., 2013; Tristezza et al., 2013; Legras et al., 2018; Peter et al., 2018). This biodiversity is strictly associated with a significant high technological variability (Pretorius, 2000) and is of great importance for a successful strain selection and the development of new starters able to modulate the organoleptic quality of wine (Romano et al., 2003).

Wild strains of *S. cerevisiae* are genetically and phenotypically distinguished from the selected commercial starter strains that are the result of selection programs (Peter et al., 2018; Pontes et al., 2020). In general, the commercial strains are characterized by high ethanol and low-pH tolerance, and they exhibit scarce production of aromatic compounds and low sporulation activity and biodiversity level (Duan et al., 2018; Kang et al., 2019). On the contrary, the wild strains, possessing high genotypic and phenotypic diversity, produce relatively high amounts of different secondary metabolites, thus offering considerable potential for utilization in industrial applications (Kang et al., 2019). Therefore, wild isolates from flowers and sugar-rich sources can lead to an aromatic profile characterized by specific volatile compounds capable of characterizing wine (Pontes et al., 2020; Alfonzo et al., 2021). As an example, wine fermentations using native wild strains obtained from oaks produce earthy and sulfurous organoleptic characteristics but intense of citrus and floral attributes (Hyma et al., 2011).

Thus, although industrial yeast strains represent a fundamental tool for reproducing the final quality of table wines, their massive use is not recommended for traditional wines in which peculiar traits are desired (Spano et al., 2010; Capozzi and Spano, 2011). For these reasons, indigenous yeast starters, which are supposedly well adapted to a specific grape must and reflect the biodiversity of a particular “terroir” are more and more requested by winemakers (Bokulich et al., 2014; Gilbert et al., 2014; Feghali et al., 2020). Indeed, it is hypothesized that in different vitivinicultural regions, specific yeast strains are naturally selected and that they are able to exalt the sensorial and aromatic profile of wine produced in that area. In fact, Knight and Goddard (2015) showed that genetically differentiated population of *S. cerevisiae* in New Zealand had a different impact on wine quality due to the production of a different complex mix of chemicals. Setati et al. (2012), while studying the spatial distribution of fungal microbial communities within and between vineyards from the same “terroir” found higher yeast heterogeneity on grape samples collected at different points inside individual vineyards than between vineyards with very contrasting farming strategies. Thus, the myriad of microclimates occurring within each vineyard due to differential shading of grapes by leaves, and the aspect of each grape cluster, greatly affects the qualitative/quantitative composition of the vineyard-associated yeast microbiota. Bokulich et al. (2014) used a high-throughput short-amplicon sequencing approach to demonstrate that specific regional and grape-variety factors shape the biodiversity of fungal and bacterial consortia inhabiting wine-grape surfaces. Indeed, the microbial assemblages correlate with specific climatic features, and this suggests a link between vineyard environmental conditions and microbial residence patterns. Taken together, these findings reveal the importance of microbial populations for the regional identity of wine (Bokulich et al., 2016) and underline that the utilization of *S. cerevisiae* indigenous strain with selected traits is fundamental to modulate the final characteristics of the wine.

The first step toward the attainment of indigenous *S. cerevisiae* wine starters is the clonal selection of the yeast strains associated with the wine-producing area of interest. Clonal selection is based on the evaluation of a number of phenotypic characteristics that are requested to guarantee the production of wines with peculiar sensorial properties. Traditionally, these are distinguished in technological and qualitative characteristics. Technological characteristics, such as fermentation power (ethanol production), fermentation purity (grams of acetic acid produced/100 ml ethanol), fermentation ratio, SO_2_ resistance, dominance on wild microbiota, and others, affect the progression and efficiency of grape must fermentation. Qualitative characteristics, among which enzymatic activities, production of fermentation-associated metabolites (glycerol, acetaldehyde, succinic acid, pyruvic acid, higher alcohols, etc.) modulate the chemical and sensorial profile of wine.

Indeed, the expression of all these characteristics can be distorted by the experimental conditions adopted for their evaluation, thus leading to inconsistent results. In this context, the definition of a standard protocol aimed at obtaining a representation of the enological performance of a yeast strain appears to be of great importance, as it would allow an unbiased comparison among strains.

Therefore, the Italian Group of Microbiology of Vine and Wine (GMVV), belonging to the Scientific Society SIMTREA,^1^ developed a common experiment for the characterization of wine strains belonging to *S. cerevisiae* species. The GMVV, including different research units (RUs) from all Italian Universities and Research Centers, has the mission to collect the skills concerning wine microbiology with the main purpose to build a benchmark for science and wine industry, able to offer applicative solutions. Therefore, in order to evaluate the extent of experimental differences due to the fermentation carried out in different laboratories with the same yeast strain, 17 RUs used two commercial *S. cerevisiae* strains (EC 1118 and AWRI796) to perform inter-laboratory-scale comparative fermentations using both synthetic medium and grape musts and applying the same protocol to obtain reproducible and statistically valid results.

## Materials and Methods

### Yeast Strains

Two widely used commercial *S. cerevisiae* active dry yeast (ADY) strains were used in this study: Lalvin EC1118 (Lallemand Inc., Montreal, QC, Canada), coded as EC, and AWRI796 (MAURIVIN, Tebaldi, Varese, Italy), coded as AW. In order to use the same batch of each commercial yeast for all the tests performed in the different laboratories, one RU prepared and sent sterile Falcon tubes containing the ADY strains to the other RUs.

### Synthetic and Grape Musts

The tests were performed in synthetic must by three RUs (coded as A, B, and C) and in grape must by eight RUs (coded from D to K), following the same protocol of media preparation, strain inoculation, and the fermentation process control.

According to Table 1 of OIV-OENO 370-2012 resolution (Anonymous, 2012), the synthetic must contained 200 mg/L of assimilable nitrogen and 230 g/L of sugar. The medium was sterilized by 0.2-μm membrane filtration (Pall Corporation, Port Washington, NY, United States). Table 1 describes the steps of the common procedure followed for the experiment in synthetic must.

**TABLE 1:** Protocol for standardization of fermentation trials in synthetic must carried out by *Saccharomyces cerevisiae* EC1118 and *S. cerevisiae* AWRI796.

Step	Procedures
Yeast strain supply	The active dry yeast (ADY) belonging to the same lot should be used
Synthetic must preparation	The synthetic must composition is reported in Table 1 of the resolution OIV-OENO 370-2012 (Anonymous, 2012)
Synthetic must distribution	500-ml Erlenmeyer flasks containing 350 ml of synthetic must and equipped with Muller valves are used. The trials are carried out in triplicates (three independent experiments)
Yeast rehydration	According to the resolution OIV/ENO 329/2009 (Anonymous, 2009), each ADY strain is rehydrated as follows: • weigh 1 g of ADY under aseptic conditions; • add 100 ml of 5% sucrose solution in water at 36–40°C under sterile conditions; • homogenize slowly using a rod or a magnetic stirrer for 5 min; • stop stirring and allow to stand for 20 min at a temperature between 36 and 40°C; • homogenize again at room temperature for 5 min; • take 10 ml under sterile conditions and then proceed to count viable yeast cells by Thoma counting chamber using 0.1% (w/v) methylene blue solution.
Yeast strain inoculum	Inoculate the rehydrated yeast in the synthetic must in order to get 2 × 10^6^ cells/ml
Fermentation trial conditions	Incubate the Erlenmeyer flasks closed with Muller valves (containing sulfuric acid) at 25 ± 2°C in static conditions for 15 days
Fermentation monitoring	Check the weight loss daily after shaking each Erlenmeyer flask by hand for 1 min
Sample arrangement for analyses	At the end of the fermentation, centrifuge at 3,000 × *g* for 5 min at room temperature and separate the cells from the supernatant
Chemical analyses	Resulting wines should be analyzed at certified laboratory by official OIV methods.

Regional grape musts, obtained from red or white grapes, were used for trials from each RU involved in this phase of the work (Table 2). In order to standardize yeast assimilable nitrogen (YAN) of each grape must, this parameter was previously measured and adjusted to a final content of 7.5 mg/L of YAN per 10 g/L of initial sugars. For this purpose, a stock solution of 10 g/100 mL of “Supervit” (Enartis, Novara, Italy) corresponding to 20 mg/mL of YAN was used.

**TABLE 2:** Main enological parameters of grape musts used from the research units (RUs).

Must from RU	Ethanol (%v/v)	Glucose (g/L)*	Fructose (g/L)*	Glycerol (g/L)	pH	Volatile acidity (acetic acid g/L)	Grape variety**
RU-D	<0.10	86	85	<0.50	3.19	0.05	Sangiovese (R)
RU-E	<0.10	104	106	<0.50	3.25	0.05	Magliocco (R)
RU-F	<0.10	112	112	<0.50	3.85	0.08	Nerello/Magliocco (1:1) (R)
RU-G	<0.10	110	115	<0.50	3.06	0.05	Barbera (R)
RU-H	<0.10	118	120	0.70	3.16	0.05	Cabernet Sauvignon (R)
RU-I	<0.10	111	112	0.80	3.42	0.07	Negroamaro (R)
RU-J	<0.10	100	98	<0.50	3.50	0.07	Moscato (W)
RU-K	<0.10	90	96	<0.50	3.32	0.08	Incrocio Manzoni (W)

*The precision of the method does not allow the use of decimals.

**R, red; W, white.

Different volumes of potassium metabisulfite (stock solution, 10 g/L) were added to each grape must, according to its pH, in order to obtain 20 mg/L of SO_2_.

### Yeast Inoculum Preparation

Each ADY strain was rehydrated according to the method described in the resolution OIV OENO 329/2009 (Anonymous, 2009). In detail, 1 g of ADY under aseptic conditions was suspended in 100 mL of a 5% sucrose solution in water at a temperature between 36 and 40°C. The suspension was slowly homogenized for 5 min with a rod or a magnetic stirrer, left to rest for 20 min at a temperature between 36 and 40°C, homogenized again for 5 min at room temperature. Finally, the homogenized yeast solution was subjected to the Thoma hemocytometer chamber count to assess the yeast cell density, and an adequate volume was inoculated in the flasks in order to reach 2 × 10^6^ cell/mL.

### Fermentation Trials

The fermentations were carried out in 500-mL flasks filled with 350 mL of synthetic or regional grape must. The tests were performed in triplicate (each strain in 3 flasks = 6 fermentation trials per each RU). After inoculating, the flasks with the must were closed with Muller valves (containing sulfuric acid up to the height of the internal glass tube) and incubated at 25 ± 2°C in static conditions both in synthetic and regional musts to gain reproducible results; fermentation lasted 15 days in synthetic must and until sugar exhaustion (<2.0 g/L) for the regional grape musts. Fermentation kinetic was determined by the weight loss of samples during the process (Castelli, 1954; Massera et al., 2012). Weight loss was measured daily after shaking by hand for 30–60 s each flask. At the end of the fermentation, the samples were centrifuged at 3,000 × *g* for 5 min at room temperature in order to separate yeast cells from the supernatant. The resulting cells from each RU were washed with sterile distillate water and centrifuged using the above-mentioned conditions, and the collected cells were transported at 4°C to the RU-L for the metabolomic analysis. In order to reduce the analytical variability of different instruments, the resulting supernatants from synthetic and regional fermented musts, collected from each RU, were sent to the RU-M for the analysis of the volatile components and to an accredited laboratory (ISVEA s.r.l., Poggibonsi, Siena, Italy) for chemical analysis.

### Chemical Analyses

Synthetic and regional fermented grape musts were analyzed at the ISVEA s.r.l. laboratory (Poggibonsi, Siena, Italy) by official OIV methods (Anonymous, 2019) to determine the concentrations of the following parameters: ethanol, glucose, fructose, and glycerol by high-performance liquid chromatography (HPLC) (Agilent 1200 Series HPLC System; Agilent Technologies Italia S.p.A., Cernusco sul Naviglio, Italy) and higher alcohols, ethyl acetate, and acetaldehyde by gas chromatography (Agilent 7890 Gas Chromatograph System) and pH values by using a pH-meter. The chemical composition of unfermented grape musts was also assessed. ISVEA is an analysis laboratory authorized by the Italian Ministry of Agriculture, Food, and Forestry Policies for wine certification and accredited by “ACCREDIA”.

### Volatile Molecule Profiles of Wines

The volatile molecule profiles of the obtained wines were analyzed using a gas-chromatographic/mass spectrometry (GC/MS, Shimadzu QP2010, Shimadzu, Kyoto, Japan) coupled with the head-space solid phase microextraction (HS-SPME) technique. For each sample, 5 mL of wine, added of 4-methyl 2-penthanol as internal standard (100 mg/L) and 0.5 g of NaCl, was sealed in a sterile vial. Samples were heated in a GC autosampler at 40°C for 10 min and volatiles adsorbed for 30 min on the fused silica fiber (CAR/PDMS, 65 μm, SUPELCO, Bellefonte, PA, United States). Molecules were desorbed in the gas-chromatograph for 5 min using a Zebron ZB-WAX 52 30 m × 0.25 μm column (Phenomenex, Torrance, CA, United States). The gas-chromatographic conditions were as follows: injection temperature, 250°C; interface temperature, 240°C; ion source, 200°C; carrier gas (He) flowrate, 2 mL/min; and splitting ratio, 1/10 (v/v). The oven temperature was programmed as follows: 40°C for 10 min; from 40 to 200°C, with a 3°C/min rate of increase; from 200 to 240°C, with a 10°C/min increase, then holding for 5 min. Volatile molecules were identified by referencing NIST 8.0 (National Institute of Standards and Technology, Gaithersburg, MD, United States). The quantification of volatile compounds in equivalent mg/L was performed on the basis of the internal standard.

### Fourier Transform Infrared Metabolomic Fingerprinting

Fourier transform infrared (FTIR) spectroscopy was performed for each 1 of the 66 samples [two yeast strains, each inoculated in both synthetic (3 RUs) and different regional (8 RUs) grape musts in triplicate]. Samples were thawed at room temperature (RT) for 30 min, and a volume of 105 μL was then sampled for three independent FTIR readings [35 μL each, according to the technique suggested by Essendoubi et al. (2005)]. FTIR measurements were performed in transmission mode. All spectra were recorded in the range between 4,000 and 400 cm^–1^ with a TENSOR 27 FTIR spectrometer, equipped with HTS-XT accessory for rapid automation of the analysis (BRUKER Optics GmbH, Ettlingen, Germany). Spectral resolution was set at 4 cm^–1^, sampling 256 scans per sample. OPUS version 6.5 software (BRUKER Optics GmbH, Ettlingen, Germany) was used to carry out the quality test, baseline correction, vector normalization, and the calculation of the first and second derivatives of spectral values.

### Statistical Analysis

The experiments were performed over three batches (independent samples) and repeated three times per batch (technical replicates or dependent samples). The basic assumption of homoscedasticity was preliminary checked for all data through the software Statistica for Windows (Statsoft, Tulsa, OK, United States).

The data on synthetic must were standardized and used as amounts per gram of consumed sugar (glucose and fructose) and then analyzed through Kruskal–Wallis ANOVA by ranks, multiple comparison by Kruskal–Wallis and chi square, and graphically reported as box–whisker plot, where the central point is the median, and the box accounts for the interquartile range and whiskers, if available, with the minimum and the maximum values. The combination “strain vs. RU” (EC-RU-A, EC-RU-B, EC-RU-C, AW-RU-A, AW-RU-B, and AW-RU-C) was used as categorical predictor. The critical value of p was set to 0.05.

Main volatile compounds (higher alcohols, ethyl acetate, and acetaldehyde) from synthetic must fermentation were also analyzed through two-way joining; for this latter analysis, the amalgamation method was based on the single linkage approach and percent disagreement.

All chemical data obtained from grape must fermentations were analyzed through two-way analysis of variance because the homoscedasticity was verified; RU and strain were used as categorical predictors, and the results were reported as table of standardized effects and pictures on the decomposition of the statistical hypothesis. p was set to 0.05. Volatile compounds were also analyzed through two-way joining (single linkage approach and percent disagreement) and principal component analysis (PCA) (Euclidean linkage approach). For two-way joining, the data of the same strain for all RUs were put together, while for PCA, the data of each RU were separately analyzed to point out strain difference.

Fourier transform infrared data were analyzed by cluster analysis algorithms, using the OPUS software (Bruker GmbH, Ettlingen, Germany). Hierarchical cluster analysis was carried out to compare the different samples using as input raw spectra, vector normalized spectra, and second derivatives spectra and considering different spectral regions. Heterogeneity within the dendrogram (reported as y-scale of the dendrogram) has been defined according to the Ward’s algorithm, using Formula 1:


Formula⁢1⁢H⁢(r,i)=⁢{[n⁢(p)+n⁢(i)]⋅D⁢(p,i)+[n⁢(i)+n⁢(q)]⋅D⁢(q,i)-n⁢(i)⋅D⁢(q,i)}/[n+n⁢(i)]

where H indicates the heterogeneity, D indicates distances, n indicates the number of spectra, subscripts “p” and “q” indicate successive clusters, whereas the “i” subscript designates the ith spectrum whose heterogeneity is calculated. Spectra were classified by using the OPUS cluster analysis based on a hierarchical classification algorithm. The procedure went as follows: vectorial normalization and the calculation of the second derivative using a Savitsky–Golay algorithm, with nine smoothing points. This pre-processing was carried out for all spectra on the spectral region with biologically relevant information (cm^–1^ [3,200 − 2,800] + [1,800 − 700]). The derivation of the spectra to the second order was used to increase the number of discriminants features present in the spectra. The spectra were classified by using the OPUS hierarchical cluster analysis based on Ward’s classification algorithm. The function used minimized the variance intra-class of the spectra and represented this in a cluster, according to their similarities. The spectral windows were chosen to obtain a consistent classification of the strains.

## Results and Discussion

### Fermentation Trials in Synthetic Must

From a practical point of view, the validation of a protocol could be divided into different steps: validation *sensu stricto*, verification, and performance evaluation (UNODC, 2009). This paper does not evaluate the first point (validation *sensu stricto*) because this challenge has been addressed by OIV when developed the current method and proposed the analytical indices (sensitivity, linearity, limit of detection, etc.). This paper focuses on the verification and on performance monitoring of the method, intended as gaining the same results when using the same conditions in different laboratories. Therefore, as a first step of the experiment, three RUs (A, B, and C) assessed the fermentative properties of the two commercial *S. cerevisiae* strains (coded as EC and AW) in a specific synthetic must; chemical analyses were done by a single certified laboratory to avoid another source of variability linked to equipment and tool calibration or to the use of in-house methods different from laboratory to laboratory (UNODC, 2009).

At the end of the alcoholic fermentation, residual sugars from 0.5 to 2.1 g/L occurred in the different trials. Therefore, a preliminary standardization of the data was done by calculating the amounts of fermentative products per unit of consumed sugar to address the prerequisites of statistic that is the comparability of datasets of different origin. Standardization is often necessary for biological systems because each system has its own traits depending, for example, from different input conditions (in this paper, the amount of consumed sugar). A different input could produce outputs that seemed to be different, but this is a statistical construct depending on the fact that the starting point is variable (Bevilacqua et al., 2016); standardization avoids these artifacts and renders data comparable.

**FIGURE 1:**
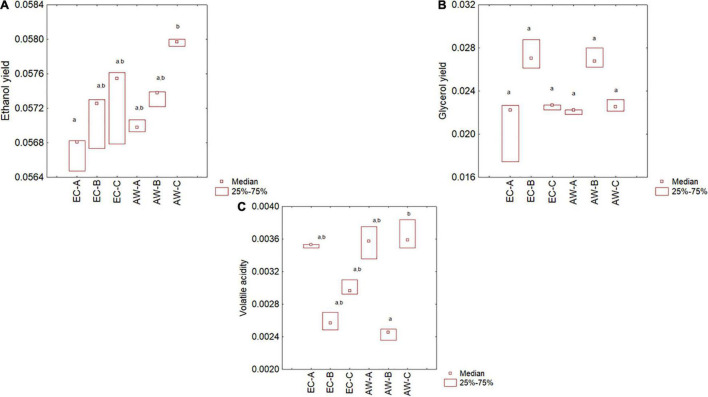
Box plots of ethanol (% v/v per unit of consumed sugar) **(A)** and glycerol yields (g/L per unit of consumed sugar) **(B)** and volatile acidity (g/L per unit of consumed sugar) **(C)** produced by the two strains (EC, AW) in synthetic must trials carried out by three RUs (A, B, and C). Different letters on plots indicate significant differences (*p* < 0.05).

Another challenge was the homoscedasticity, which was not addressed for the results gained in the synthetic medium; therefore, data were analyzed through a non-parametric test and represented through a box-plot graph. Figure 1 shows the amounts of ethanol, glycerol, and volatile acidity produced by the strains in the synthetic must.

The ethanol yield (amount of ethanol produced per unit of sugar consumed) (median) (Figure 1A) was 0.057–0.058 v/v; Kruskal–Wallis and median test highlighted that the differences amongst the different RUs were not significant (*p* > 0.05). The only difference was between the ethanol of the strain EC from RU-A and the strain AW from the RU-C (p, 0.026). Glycerol yield (g/L of glycerol produced per unit of consumed sugar) (Figure 1B) was from 0.024 to 0.027 g/L (per gram of consumed sugars), while volatile acidity (Figure 1C) was from 0.0024 to 0.0036 of acetic acid (g/L per gram of consumed sugars). For this latter parameter, a significant difference was recorded for the strain AW between the RU-B and RU-C (p, 0.043); however, from a quantitative point of view, the difference was 0.0012 g/L per gram of consumed sugar, corresponding to a global difference of 0.28 g/L for 230 g/L of sugars (concentration of glucose and fructose in the synthetic must).

**FIGURE 2:**
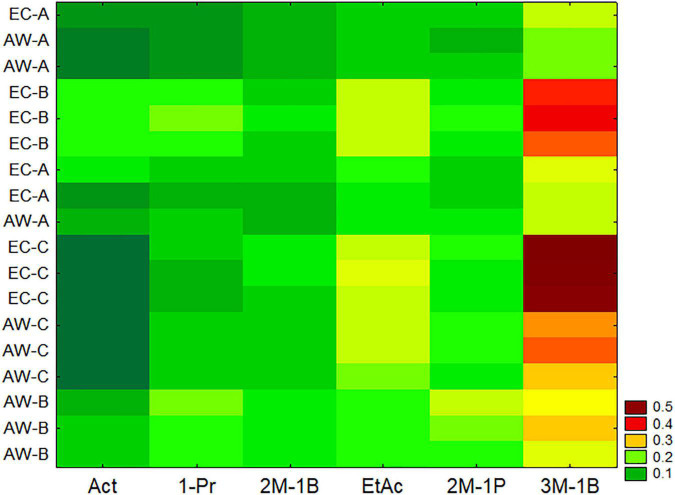
Two-way joining of the main by-products formed by the two *Saccharomyces cerevisiae* strains in synthetic must trials carried out by three RUs (A, B, and C). Act, acetaldehyde; 1-Pr, 1-propanol; 2M-1B, 2-methyl-1-butanol; EtAc, ethyl acetate; 2M-1P, 2-methyl-1-propanol; 3M-1B, 3-methyl-1-butanol; ppm per gram of consumed sugar.

Wines produced from synthetic must were also analyzed to assess the concentration of acetaldehyde, 1-propanol, 2-methyl-1-butanol, ethyl acetate, 2-methyl-1-propanol, and 3-methyl-1-butanol. Data were preliminary standardized as reported above and analyzed through a two-way joining and then by a multiple comparison (Kruskal–Wallis and median test). Two-way joining is a clustering approach with some similarities to cluster and principal component analyses; it combines cases/samples (in this research, the results for the two strains from three RUs) using a set of input variables, and the output is a clustering at global level, and also semi-quantitative results for each variable. However, it works on mean values, and for the data of this research, this could be a limit because they did not address the homoscedasticity; therefore, all batches (three independent samples for each strain per RU) were used as input cases (Figure 2).

Two-way joining highlights two important results for the aim of this research (validation of a protocol); the first one was the agreement and the similarity among the independent batches of each experiment. In fact, the batches were very close each other as a part of a single sub-cluster, with some exceptions to this statement (a batch of strain EC of RU-A and a batch of the strain AW for the same RU). Another output is the statistical tendency to put the strain EC in the upper part of the *y*-axis and the strain AW in the lower part, except for the strain AW from the RU-A.

The non-parametric test for the compounds used in the two-way joining is in Supplementary Figure 1. Generally, there were no significant differences amongst different laboratories, except for acetaldehyde, which was not recovered in the batches from the RU-C, for both the strains.

The validation of a protocol is a challenge because several variables should be considered and many factors could affect the goodness of the results and cause a systematic bias or the increase in several forms of variabilities (Ahmed et al., 2020). There are at least two strong factors to control, that are, intra- and inter-laboratories variability (Ahmed et al., 2020); both variabilities could strongly affect the quality of results.

The first kind of variability (inter-laboratory) was studied and assessed through the trials in synthetic must. Inter-laboratory variability is caused by several factors, including laboratory personnel, metrics, reliability of tools, and equipment (Scherz et al., 2017), which could be due to systematic bias; however, there is also a casual source of errors, which must be characterized and weighted because it is responsible of the uncertainty of the measure (UNODC, 2009). The causal error cannot be completely avoided, but it should be within acceptable limit because results from different laboratories should not be significantly different when using the same conditions and the same setup. This requisite is known as “replicability,” and the results from the first part addressed it because the strains showed the same trends and the RUs produced results that were not statistically different. The conditions to address replicability were the use of standardized experimental conditions and an accurate treatment of the data, including a preliminary standardization and the combination of non-parametric test and clustering approaches.

### Fermentation Trials in Regional Grape Musts

A second challenge is the reproducibility of the results, intended as obtaining consistent results across studies aimed at answering the same scientific question using new data. Generally, reproducibility is used as a synonym of replicability, but they are quite different (Plesser, 2018). Replicability, in fact, was addressed in the synthetic must because it has two main requisites: the use of the same experimental set-up by different teams (in this paper, the same strains and the same medium in three RUs). On the other hand, reproducibility relies on the use of different set-up by different teams to gain the same results (Plesser, 2018); in this paper, the result is the qualitative trends of the strains, studied by different teams and in different musts (different set-up). The challenge of reproducibility is discussed in this section and above all in the following one.

Regional grape musts (from red or white grapes) were used by each RU as fermentation substrate for the two *S. cerevisiae* strains; the trials were carried out by eight RUs (D, E, F, G, H, I, J, and K), as described previously.

As regards the main enological parameters, namely, ethanol, glycerol, and volatile acidity, the results were analyzed through two-way analysis of variance because they addressed the requisite of homoscedasticity. In addition, the use of a non-parametric test was not advisable for a high number of samples (16 samples, different for two predictors, that is strain and RU) because the variability of data could lead to bias and statistical artifact.

**FIGURE 3:**
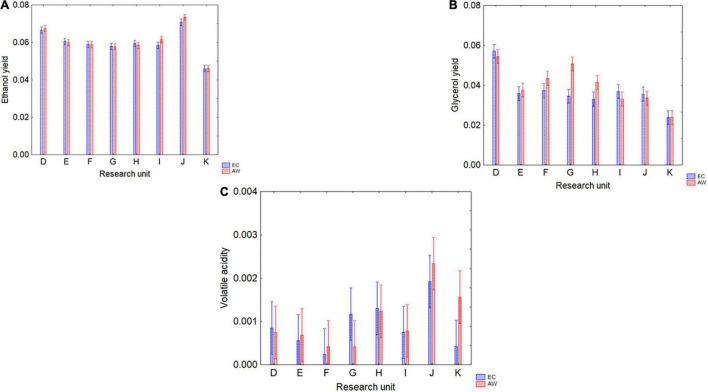
Ethanol (% v/v per unit of consumed sugar) **(A)** and glycerol yields (g/L per unit of consumed sugar) **(B)**, and volatile acidity (g/L per unit of consumed sugar) **(C)** produced by the two strains (EC, AW) in the different grape musts by eight RUs (D, E, F, G, H, I, J, and K). Decomposition of the statistical hypothesis for the interaction strain × RU; the bars indicate 95% confidence intervals.

Figure 3 shows the results for ethanol, glycerol, and volatile acidity. For ethanol yield, the only predictor playing a significant role was the RU (*p* < 0.05), while neither strain nor the interaction strain × RU were significant (data not shown); ethanol yield was from 0.045 (RU-K) to 0.070 vol/vol per gram of sugar (RU-J) (Figure 3A). Glycerol yield was from 0.024 (RU-K) to 0.057 g/L per gram of sugar consumed (RU-D) (Figure 3B), and the differences were affected by both RU and the interaction RU/strain. Finally, volatile acidity was only affected by the RU, with amounts ranging from 0.0002 to 0.002 g/L of acetic acid per gram of sugar consumed (Figure 3C).

**FIGURE 4:**
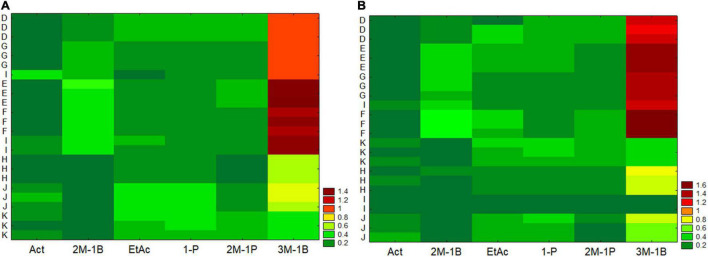
Two-way joining of the main by-products formed in different grape musts by the strain EC (graph **A**) and the strain AW (graph **B**) and by eight RUs (D, E, F, G, H, I, J, and K). Act, acetaldehyde; 1-Pr, 1-propanol; 2M-1B, 2-methyl-1-butanol; EtAc, ethyl acetate; 2M-1P, 2-methyl-1-propanol; 3M-1B, 3-methyl-1-butanol; ppm per gram of consumed sugar.

As regards the analysis of the main by-products (acetaldehyde, ethyl acetate, and higher alcohols), two-way joining was also used. For figure readability, the strains were separately analyzed, while the effect of the two strains was then assessed through a multifactorial analysis of variance. Generally, two-way joining confirmed the suitability and the accuracy of the protocol, as the independent batches analyzed per strain were clustered close to each other with the single exception for a batch from the RU-I (Figure 4). In addition, the analysis suggests the existence of different trends and amounts as a function of the RU for at least two compounds, that is, 3-methyl-1-butanol and 2-methyl-1-butanol.

Two-way joining suggests a low intra-laboratory variability within each RU. Intra-laboratory variability is linked to several factors and generally could not be controlled because it is a strong source of error and is responsible of failing of some protocols (Ahmed et al., 2020). In this research, the clustering of the replicates from each RU in the same region of *y*-axis suggests that the protocol had a low intra-laboratory variability, probably due to the application of standardized conditions and a common flowsheet, as detailed in the section “Materials and Methods”. In addition, this analysis suggests the importance of performing a robust yeast characterization, which is not possible through data produced in a single laboratory (Canelas et al., 2010).

Concerning the effect of a different must on the production of different compounds, the description of the character “must” is outside the scope of this paper; it is well known that different musts lead to different wines, depending also on the strain. However, a brief description of the qualitative trends for each compound is shortly described in the following lines.

The differences amongst the RU-grape musts and the strains were analyzed through two-way ANOVA (Supplementary Figure 2). For each compound, three different outputs were analyzed: (i) the table of standardized effects to assess the significance of each predictor (strain and RU-must) and of interactive term (strain × RU-must); (ii) the decomposition of the statistical hypothesis (bar diagram showing the quantitative results); and (iii) the table on the homogeneous groups that shows differences and similarities.

Generally, the predictor strain was never significant as individual term, but it played a significant role in interaction with the RU-must; on the other hand, RU-must was also significant as individual term. Moreover, it was the most significant term for all compounds (data not shown).

For 2-methyl-1-propanol, *post-hoc* test highlighted a continuous distribution of data with an overlapping of statistical groups: however, there were three classes. The first one comprised the wines from RUs K, J, and H (for both the strains) and AW for RU-I; they were characterized by a low concentration of 2-methyl-1-butanol (0.06–0.13 ppm/g of consumed sugar). The second class (high production of 2-methyl-1-butanol, 0.46 ppm/g of consumed sugar) contains the strain AW for the RU-F. Between these two classes, statistics pointed out an intermediate class (or transition class), composed of batches belonging to two or more homogeneous groups (wines from RUs D, G, and E for both strains and RU-I for the strain AW and wine RU-F for the strain EC) with a concentration of 2-methyl-1-butanol ranging from 0.17 to 0.40 ppm/g of consumed sugar (Supplementary Figure 2.1).

A continuous distribution was also found for 3-methyl-1-butanol (Supplementary Figure 2.2), with three classes (low-production, transition, high production). In the low-production class, there were the wines from RUs K, H, and J (for both the strain) and AW/RU-I (3-methyl-1-butanol at 0.32–0.69 ppm/g of consumed sugar), whereas the high-production class was composed of the wines from RUs E and F for both the strains, and EC/I, AW/D, and AW/G (3-methyl-1-butanol at 1.19–1.64 ppm/g of consumed sugar).

For the other compounds (acetaldehyde, ethyl acetate, 1-propanol, and 2-methyl-1-propanol), a continuous distribution of statistical groups was also recovered, but the distinction in classes was less evident (from Supplementary Figures 2.3–2.6).

### Volatile Molecule Profiles of Wines

The wines obtained from each unit were analyzed at the end of the fermentations by means of SPME/GC-MS. This technique allowed the identification of about 150 molecules belonging to different chemical classes, including principally alcohols, esters, organic acids, aldehydes, ketones, terpenic, sulfur, and other minor compounds. This technique was used since, according to the literature, it is able to provide a volatile molecule fingerprinting of food and beverages in relation to their microbiota and/or production processes (Patrignani et al., 2017). As general consideration, in all the samples analyzed (3 independent trials × 2 repetitions × each RU), independently on the initial must, the wines obtained by the strain AW were characterized by a higher presence of alcohols and esters with respect to the wines obtained from the strain EC with few exceptions (data not shown). Among alcohols, independently on the strain used, the most abundant and recorded in all the samples were 3-methyl-1-butanol (astringency, solvent aroma) 2-methyl-1-propanol (alcohol aroma), and phenylethyl alcohol (rose aroma), while for esters,1-butanol 3-methyl acetate (banana and pear aroma), ethyl acetate (fruit and solvent aroma), ethylic esters of fatty acids (grape, apple, and pineapple aroma). However, other minor alcohols (especially terpenic ones) and esters (butanoic acid esters, acetic acid esters) were detected in the volatile aroma profiles of the analyzed samples. Among acids, acetic acid, octanoic, hexanoic, and decanoic ones were the most abundant.

**FIGURE 5:**
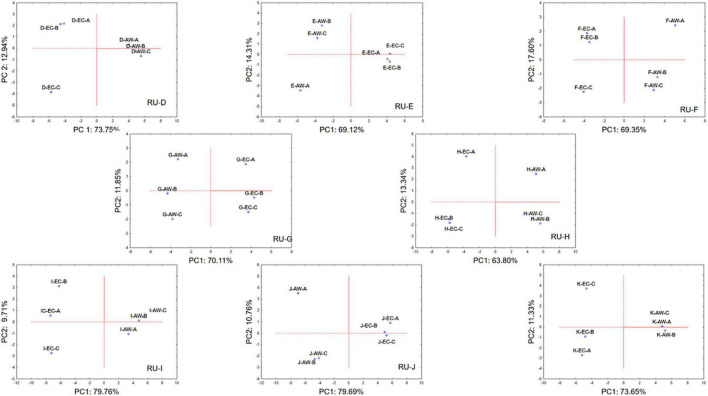
Principal component analysis plot based on volatile metabolites produced by *S. cerevisiae* EC1118 (EC) and *S. cerevisiae* AWRI 796 (AW) strains in grape must fermentations carried out by each Research Unit (RU) on volatile metabolites. Case projection, variable projection is in Supplementary Figure 3.

Due to the large dataset of information acquired, the raw volatile compound data coming from the wines obtained from each RU were analyzed by means of PCA in order to pinpoint the effects of the strains AW and EC. Figure 5 (and the Supplementary Figure 3, for variable projection) describes the PCA loading plot of volatile molecules in relation to the two strains employed and the initial used regional must in the space spanned by the first two principal components (PC1 and PC2). For each RU and wines obtained using the two *S. cerevisiae* strains, the figures report the projection of the three independent trials (each one as mean of two technical repetitions).

In general, for each wine obtained from the same initial must but using the two different *S. cerevisiae* strains, PC1 was able to describe most of the variance detected among the samples with values ranging from 65 to about 80%, while PC2 described the lowest variance with values from about 11 to 17%. As clear from the obtained figures, independently from the initial must, the wines produced by the strain EC were well separated, along PC1, from wines produced by the strain AW. Moreover, for each wine considered starting from the same must and the same strain, the three independent samples were well grouped, since PC1 was able to describe most of the variance among the samples with respect the PC2.

PCA confirmed the suitability of the method in terms of replicability because the two strains always behaved in a different way, also using a different must; moreover, it also stressed the low-intra-laboratory variability because the three replicates were always in the same region of the factorial space (Canelas et al., 2010).

Wines obtained by the different RUs, using the same *S. cerevisiae* strain, were generally characterized by different qualitatively volatile molecule profiles in relation to the must employed, contrarily to the results obtained from all the three RUs using the same fermentation agent in synthetic must. In fact, it is well known that the formation of volatile compounds during grape must fermentation depends on several factors, including the nature and concentration of the precursors initially present in the must (their proportions differ from one grape variety to another), the capacity of the naturally occurring or inoculated yeasts to transform them, and the conditions used in winemaking process, including aging (Roldán et al., 2021).

A detailed description of the effect of the most important metabolites on the spatial distribution of the strain is in Supplementary Material 4.

### Fourier Transform Infrared Metabolomic Fingerprinting

**FIGURE 6:**
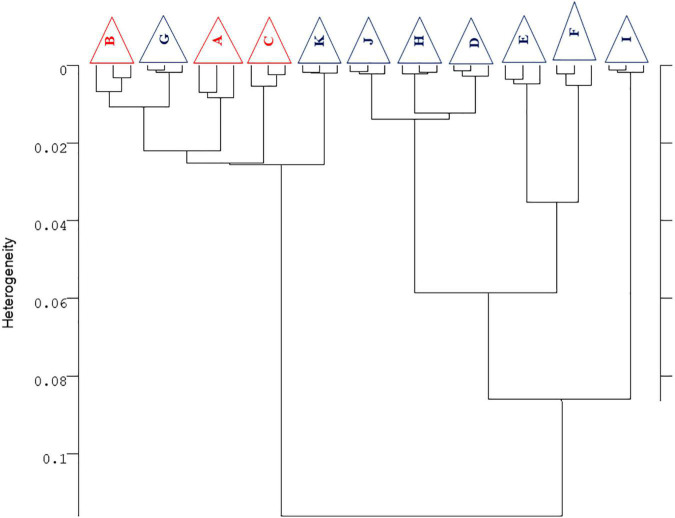
Hierarchical cluster analysis of FTIR second derivative normalized spectra obtained from the yeast strain AWRI 796 (AW) inoculated in grape must (red samples) and synthetic must (blue samples) in eleven different trial sites. The dendrogram was obtained by calculating the Euclidean distance between whole IR spectra. For each RU, three biological replicates were considered (displayed by a triangle when the replicates clustered together).

Figure 6 shows the metabolomic profiles of AW strain for both synthetic and regional musts. Synthetic musts were grouped in the same cluster, thus confirming the similarities of the overall profiles of the strain AW employed by the different RUs at global level and not only for each compound, as reported in the previous sections, thus confirming the results in the term of inter-laboratory variability and robustness of the protocol, as detailed previously. Concerning the results on grape musts, FTIR profiles confirmed the differences due to the different raw material, with at least four main trends: RU-G and RU-K (cluster 1); RU-J, RU-H, and RU-D (cluster 2); RU-E and RU-F (cluster 3); and RU-I (cluster 4).

Another important result in terms of protocol goodness is the fact that the independent batches of each fermentation cluster were put close each other, thus confirming the reproducibility of the experiments performed in the same RU. The metabolomic profiles of the strain EC do not confirm what has been described for the strain AW (data not shown). More than the ineffectiveness of the technique, this is attributable to some problems that occurred during the sampling, transport, or freezing phase of the samples. FTIR spectroscopy, in fact, is an extremely sensitive technique able to detect even the slightest variation regarding the chemical or the biological composition of the sample under study.

## Conclusion

The joint experiments, promoted by the Italian Group of Microbiology of Vine and Wine (GMVV), exploiting inter-laboratory fermentations using both synthetic medium and grape must, applying the same protocol, obtained reproducible and statistically valid results. Furthermore, the experiment proved that through integrative analysis of inter-laboratory data sets, it is possible to validate a fermentation protocol suitable for the characterization of the enological properties of wine yeasts, which would not be possible from a data set produced by a single laboratory.

Mainly, this paper has set some milestones and is a kind of forerunner for protocol validation. The key findings could be summarized as follows:

(a) The first step for a protocol validation is the assessment of replicability, hereby proposed through the trials in the synthetic must. However, this step has two main requisites: the use of a standard protocol in different laboratories and the combination of both parametric and non parametric tests, coupled with data standardization. In fact, the use of a standard protocol alone is not enough to gain reproducible and similar results; this research also pointed out that data treatment is a critical step for replication to avoid artifact and to gain robust conclusions.
(b) The second step for a validation of a protocol for microbiological purposes, such starter characterization, should also assess on reproducibility, intended as the ability to recover similar qualitative trends for microorganisms also in different matrices. Reproducibility was studied through the fermentation trials in grape musts, thus always gaining the same differences between the two strains. For this second step, the requisites are again the use of a standard protocol and a proper data treatment to compare results from different biological systems.

In conclusion, the validation of a protocol for starter characterization for enological purposes is possible, and the method hereby proposed is suitable, but a rigorous coupling between laboratory experiments and data treatment is required for a robust and effective process.

## Data Availability Statement

The raw data supporting the conclusions of this article will be made available by the authors, without undue reservation.

## Author Contributions

PR and LG contributed to the conception and design of the study and organized the database. AB performed the statistical analysis. PR, LG, and AB wrote the first draft of the manuscript. FP, MC, LC, MB, and IM wrote the sections of the manuscript. FG, VCa, KR, and VE contributed to the manuscript revision. All authors performed the laboratory experiments and read and approved the submitted version.

## Conflict of Interest

The authors declare that the research was conducted in the absence of any commercial or financial relationships that could be construed as a potential conflict of interest.

## Publisher’s Note

All claims expressed in this article are solely those of the authors and do not necessarily represent those of their affiliated organizations, or those of the publisher, the editors and the reviewers. Any product that may be evaluated in this article, or claim that may be made by its manufacturer, is not guaranteed or endorsed by the publisher.

## Footnotes

1
https://www.simtrea.org/
