# Supplementary material for: Validation of a Standard Protocol to Assess the Fermentative and Chemical Properties of Saccharomyces cerevisiae Wine Strains
Source: Front Microbiol. 2022 Feb 23;13:830277. doi: 10.3389/fmicb.2022.830277 (PMC8963721; doi:10.3389/fmicb.2022.830277)
Supplement: Supplementary file 1 [file Data_Sheet_1.docx]

Validation of a standard protocol to assess the fermentative and chemical properties of *Saccharomyces cerevisiae* wine strains

Romano et al.

**SUPPLEMENTARY MATERIAL**

**Figure S1.** Box plots of acetaldehyde, ethyl acetate, 1-proponal, 2-methyl-1-propanol, 2-methyl-1-butanol, and 3-methyl-1-butanol produced by the two strains (EC, AW) in synthetic must trials carried out by three RUs (A, B, C). Different letters on plots indicate significant differences (p < 0.05).


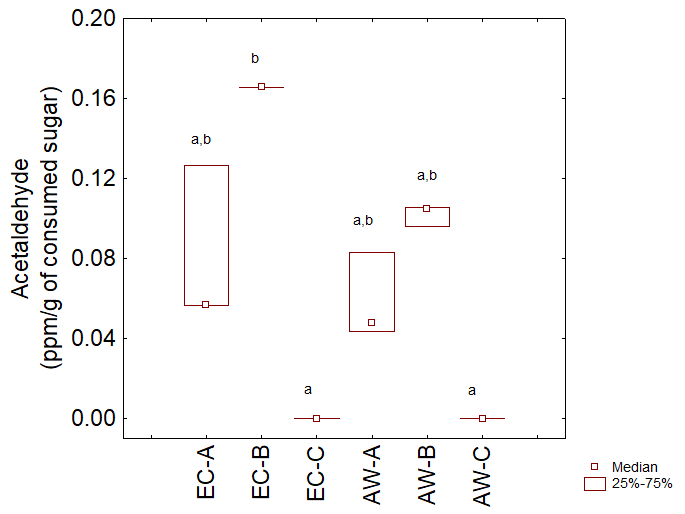

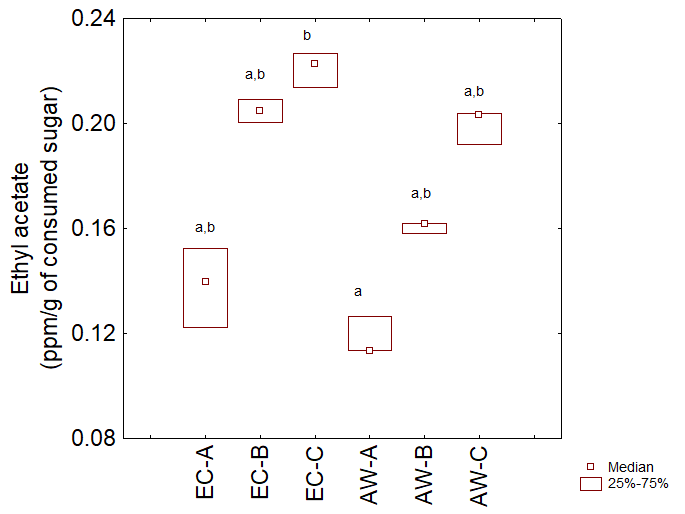


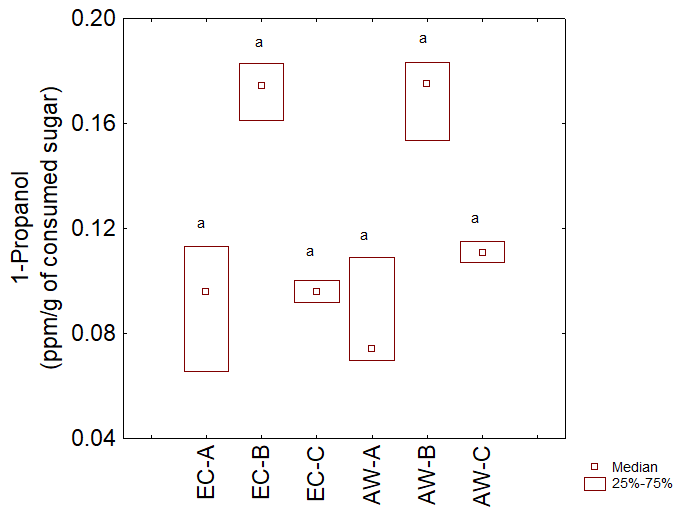

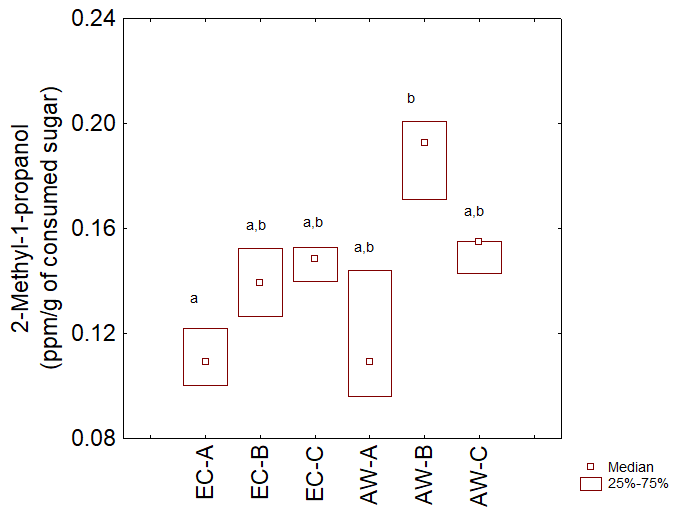


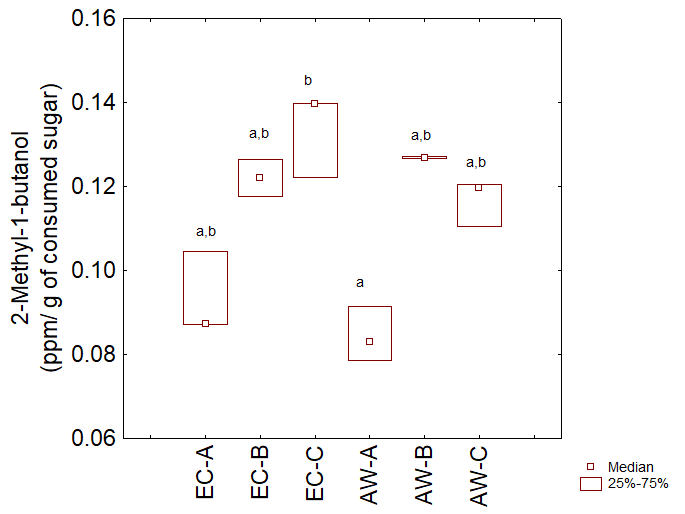

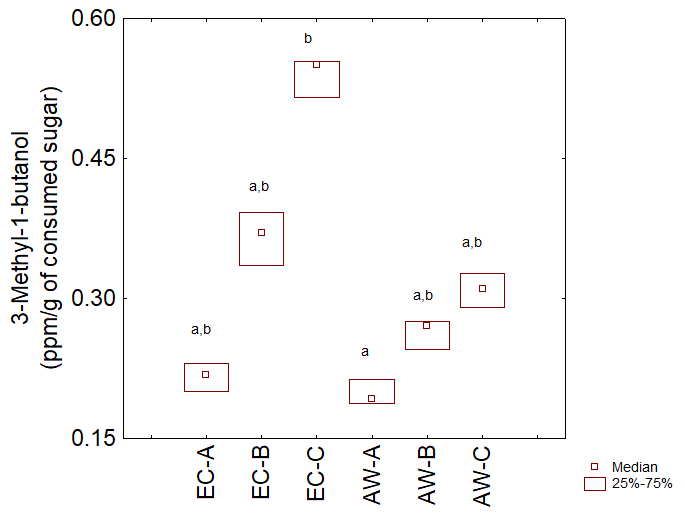


**Figure S2.** Decomposition of the statistical hypothesis (interaction RU x Strain), table of statistical effects and distribution in Statistical homogeneous groups for 2-methyl-1-propanol (S2.1), 3-methyl-1-butanol (S2.2), acetaldehyde (S2.3), ethyl acetate (S2.4), 1-proponal (S2.5), 2-methyl-1-butanol (S2.5). Two way-ANOVA and Tukey’s test.

S2.1


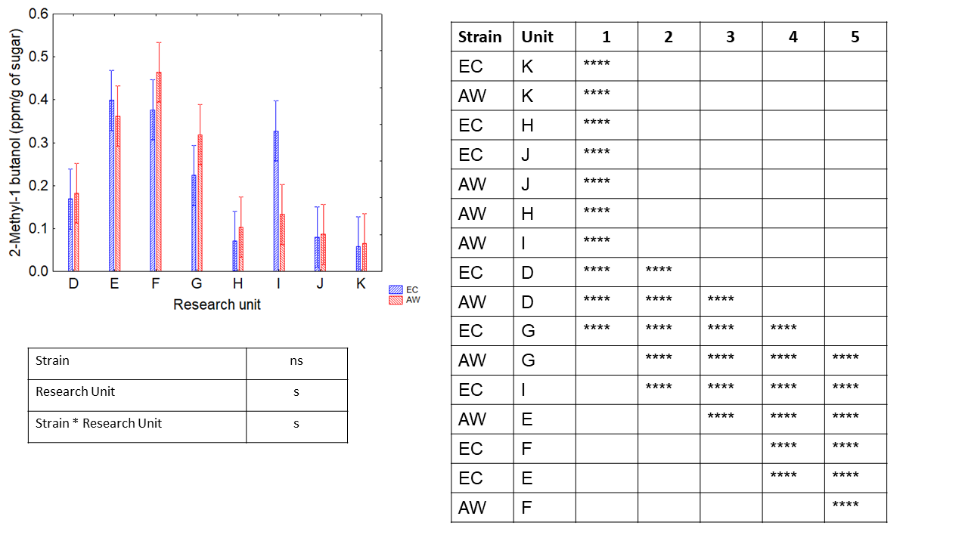


S2.2


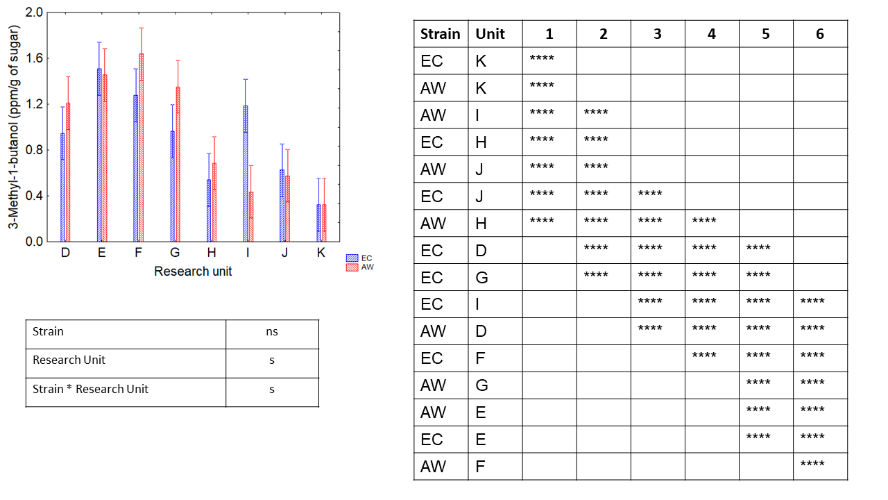


S2.3


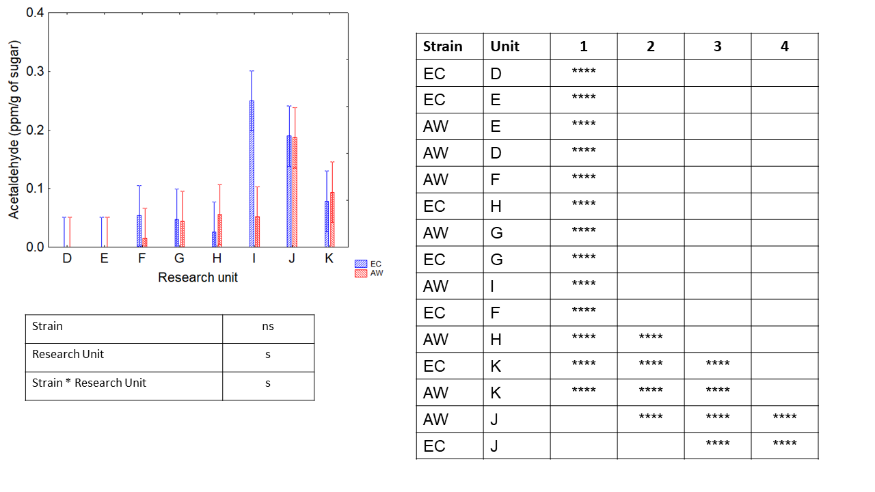


S2.4


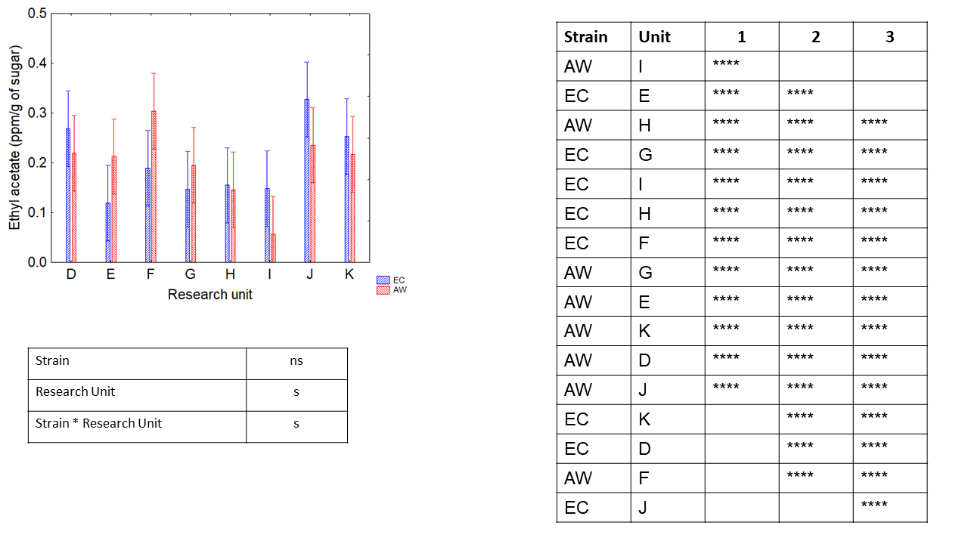


S2.5


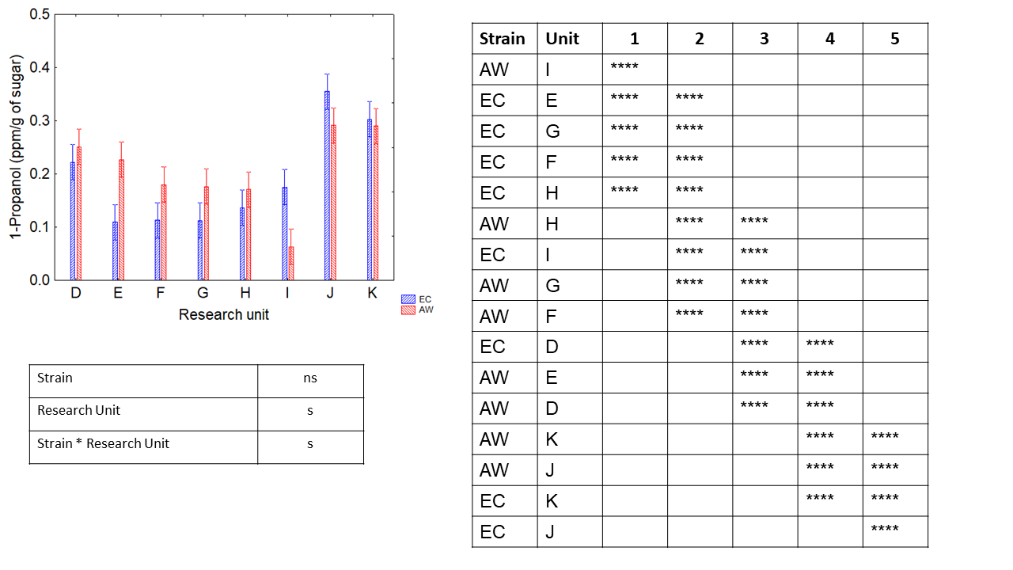


S2.6


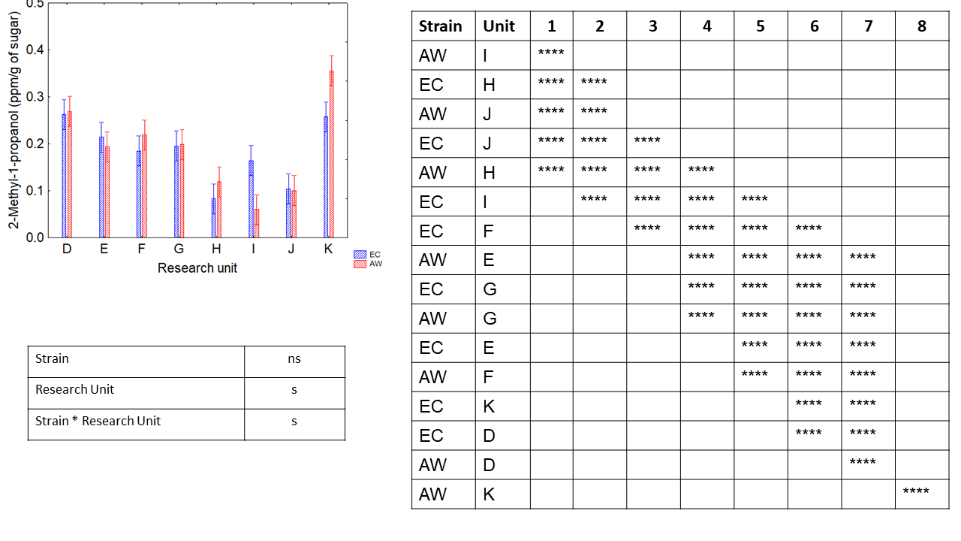


**Figure S3.** Principal Component Analysis plot based on volatile metabolites produced by *S. cerevisiae* EC and *S. cerevisiae* AW strains in grape must fermentations carried out by each Research Unit (RU) on volatile metabolites. Variable projection; case projection is in Figure 5.


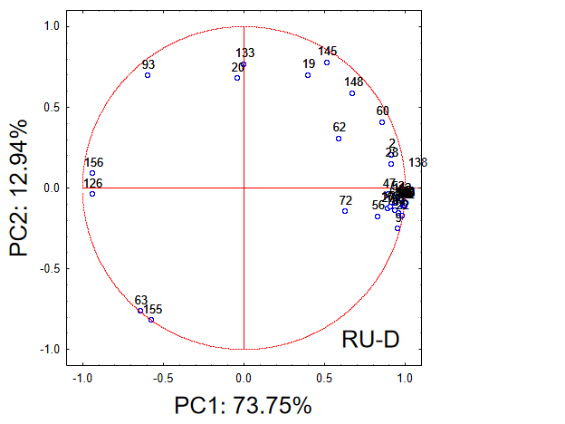

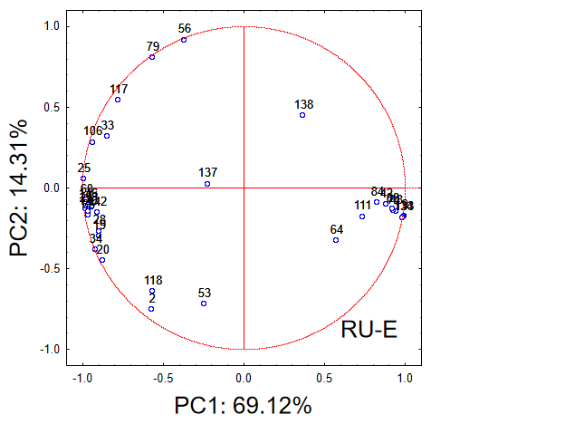


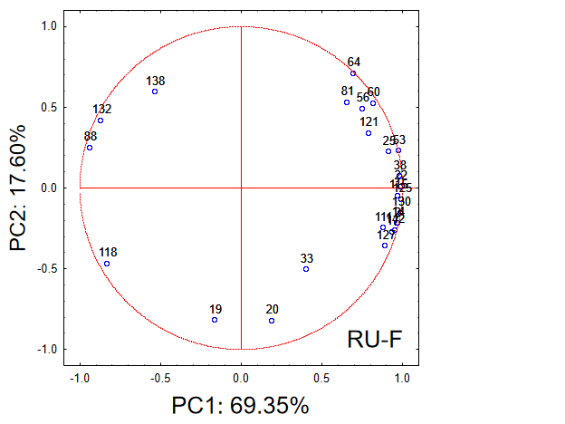

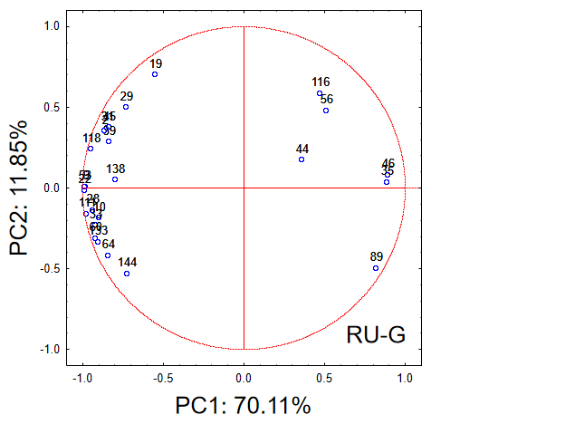


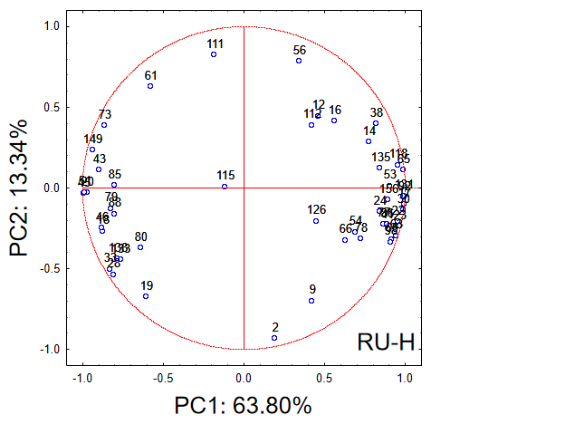

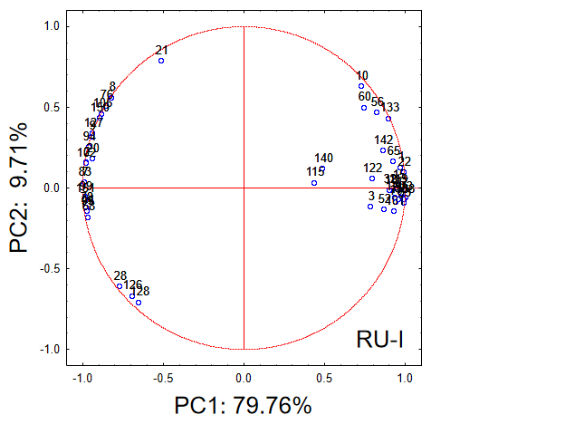


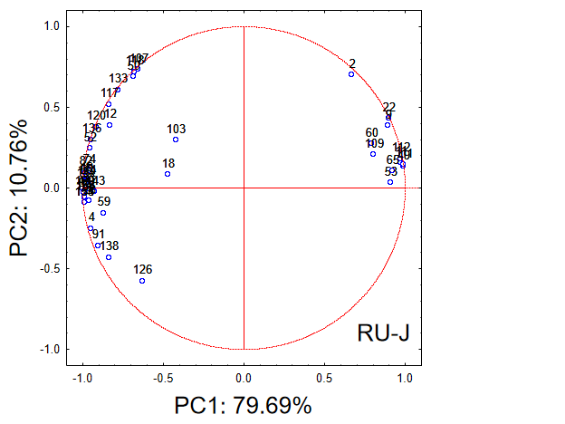

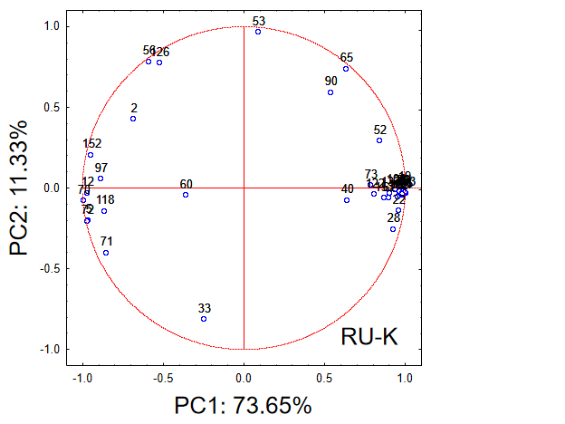


**Supplementary S4: Detailed description of PCA-Regional musts**

PCA profiles (cases and variables projections) are in Figure 5; in this section there is a detailed description of each profile to point out the contribution of the different compounds on space loading of the biological replicate.

More specifically, for the **RU-D**, the wines obtained from the two fermenting strains were well separated among PC1 able to describe the 73,75% of variance among samples. In particular, the wines from *S. cerevisiae* AW were characterized by the presence of 2-butenal, 2-methyl- (45), benzaldehyde (148), decanoic ethyl ester (19), 1-propanol, 2-amino (62), 1-propanol (60), 1-butanol 3 methyl acetate (2), hexanoic acid ethyl ester (28) while the samples from *S. cerevisiae* EC were characterized for 3-hexanol, 2,4-dimethyl (93), dodecanoic acid ethyl ester (20), 4-heptanone, 2,6-dimethyl (156), acetic acid (126), 1-propanol, 2-ethoxy (63), 3-undecanone (155).

The separation of the samples from EC and AW was detected also for samples from **RU-E** where PC1 described the 69.12% of variance among samples while PC2 just 14.31%. The molecules which permitted the grouping were 1-propanol, 2-methyl- (64), ethoxy ethanol (111), 2-propyl-tetrahydropyran-3-ol (84), tetradecanoic acid ethyl ester (42) for wines from EC while a large gamma of compounds affected the profiles of wines from AW: 1-hexanol (56), 2-propanol, 1-(1-methylethoxy) (79), 4,6-2 methyl phenol (117), octanoic acid ethyl ester (33), benzene propanol (106), octanoic acid methyl ester (34), dodecanoic acid ethyl ester (20), phenylethyl alcohol (118), 1-butanol, 3-methyl acetate (2), 1-butanol, 3-methyl (53), hexanoic acid ethyl ester (28) and 2,6 octadien 1-ol, 3,7 dimethyl acetate (3).

Considering the **RU-F**, the PC1 was able to explain about 70% of variance and the samples from EC were principally affected from the presence of octanoic acid (138), heptanoic acid (132), 3,6,9,12-tetraoxatetradecane-1,14-diol (88), phenylethyl alcohol (118), decanoic acid ethyl ester (19) while the highest presence of compounds affect the mapping of samples from AW: dodecanoic acid ethyl ester (20), octanoic acid ethyl ester (33), acetic acid, hydroxy (127), ethoxy ethanol (111), butanoic acid, 2-methyl (130), 4-hydroxybutyric acid (125), propanoic acid,2 hydroxy ethyl ester (38), formic acid hexyl ester (25), 1-butanol, 3-methyl (53), 2-hydroxybutanoic acid (121), 1-hexanol , (56), 1-propanol (60), 2-propanol, 1,1'-oxy (81), 1-propanol, 2-methyl (64). A similar trend was also observed for the wines coming from the **RU-G** where the most complexity of volatile molecules were observed for the samples from the fermentation of *S. cerevisiae* AW which were separated from samples from EC along PC1 describing the 70.11% of variance. Also, the wines from **RU-H** were separated along PC1 (able to explain the 63.80% of the variance) according to the fermentation agent employed. In particular, the wines from AW were characterized by the presence of acetic acid, 2 phenylethyl ester (9), 1-butanol 3 methyl acetate (2), 2-propanol (78), 1-butanol, 4-ethoxy (54), 1 hexanol (56), 1-propanol, 3-methylthio (66), 2 ethoxy ethanol (112), acetic acid methyl ester (12), butanoic acid, 3 hydroxy, butyl ester (16), propanoic acid,2 hydroxy ethyl ester (38), butanedioic acid ethyl ester (14), hydroxy acetic acid (135), phenylethyl alcohol (118), 1-butanol, 3-methyl (53), butanoic acid 2 ethyl 1,2,3 propanetriyl ester (15), ethyl tridecanoate (24), acetic acid (126), 4-heptanone, 2,6-dimethyl (156) while the molecules affecting the clusterisation of samples from EC were principally decanoic acid ethyl ester (19), 1-oxy 2 propanol (80), 2,6-octadien-1-ol,3,7-dimthyl acetate (3), octanoic acid (138), 2,3-butanedithiol (70), 3,6,9,12-tetraoxatetradecane-1,14-diol (88) and in less extent 1,2 aminoethylamino 2-propanol (43), 2-undecanol (85), 2,6-octadien-1-ol, 3,7-dimethyl-, (Z)-(73), 1-propanol, 2-(2-methoxy-1-methylethoxy) (61), ethoxy ethanol (111).

For **RU-I**, the wines were well separated among PC1 describing the almost 80% of variance among samples while PC2 described only the 9%. In particular, the molecules that permitted the mapping were pentanoic acid, 4-methyl (140), methyl alcohol (115), 2-hydroxybutanoic acid hydrazide (122), propanoic acid, 3-hydroxy-, hydrazide (142), 1-propanol, 2-methyl-2-nitro (65), ethyl acetate (22), hexanoic acid (133), 1 hexanol (56), 1 propanol (60), acetic acid 3 hydroxy-2,2 dimthoxy propyl ester (10) and phenethyl alcohol (118) for samples from *S. cerevisiae* AW and ethyl-9-decenoate (21), 7 nonanoic acid methyl ester (8), methyl 4 methoxyacetate (30), 2-hydroxybutanoic acid hydrazide (122), hexanoic acid ethyl ester (28), acetic acid (126), benzoic acid (128), 2 propanol 1 butoxy (83) for wines from *S. cerevisiae* EC. Similar trend was described for the samples produced from **RU-J** and **RU-K** where the most important difference among variance was explained along PC1 describing, respectively, 79.69% and 73.65% of variance among samples. In particular, the wines from **RU-J** obtained with AW were mapped in the left space of the plot for the presence of acetic acid (126), octanoic acid (138), 1 pentanol 2,3 dymethyl (59), phenethyl alcohol (118), butanoic acid ethyl ester (18), 6-octen-1-ol, 3,7-dimethyl (103), mercapto acetic acid (136), methyltiophenyl acetic acid (120), acetic acid methyl ester (12), hexanoic acid (133). On the contrary, 1-butanol, 3-methyl (53), 1-butanol 3-methyl acetate (2), ethyl acetate (22), 2-ethoxy ethanol (109), 2-ethoxy etoxy ethanol (112), 1-propanol, 2-methyl-2-nitro (65) affected the projection of the wines from EC.

Finally, the clusterisation of samples obtained from the **RU-K** was affected for sample obtained by AW by 2,6-octadien-1-ol, 3,7-dimethyl (73), 1-butanol, 3-methyl (53), ethyl acetate (22), 2-hydroxybutanoic acid hydrazide (122), hexanoic acid, ethyl ester (28), 1,6-octadien-3-ol, 3,7-dimethyl-(52), 1-propanol, 2-methyl-2-nitro (65), while for EC they were 1-hexanol (56), heptanoic acid, ethyl ester (26), 1-butanol 3-methyl acetate (2), 1,4,7,10,13,16,19-heptaoxa-2-cycloheneicosanone (152), 4,6-decadien-3-ol, 2-methyl-9-(2-methoxyethoxymethoxy) (97), acetic acid, methyl ester (12), 2,3-butanedithiol (70), phenylethyl alcohol (118), 2,5,8,11,14-pentaoxahexadecan-16-ol (72), 2,3-dimercaptopropan-1-ol (71), octanoic acid, ethyl ester (33), 1-propanol (60).
